# Prevalence and Molecular Characterization of Methicillin-Resistant *Staphylococcus aureus* from Nasal Specimens: Overcoming MRSA with Silver Nanoparticles and Their Applications

**DOI:** 10.4014/jmb.2208.08004

**Published:** 2022-11-01

**Authors:** Aly E. Abo-Amer, Sanaa M. F. Gad El-Rab, Eman M. Halawani, Ameen M. Niaz, Mohammed S. Bamaga

**Affiliations:** 1 Department of Botany and Microbiology, Faculty of Science, Sohag University, Sohag 82524, Egypt; 2 Department of Botany and Microbiology, Faculty of Science, Assuit University, Assiut 71516, Egypt; 3 Department of Biology, College of Science, Taif University, P.O. Box 11099, Taif 21944, Saudi Arabia; 4 Department of Molecular Pathology, Al-Hada Armed Forces Hospital, P.O. Box 1347, HHRC 479, Taif, Saudi Arabia

**Keywords:** MRSA, nasal specimens, resistance genes, 16S rRNA, synergistic effect, AgNPs

## Abstract

*Staphylococcus aureus* is a cause of high mortality in humans and therefore it is necessary to prevent its transmission and reduce infections. Our goals in this research were to investigate the frequency of methicillin-resistant *S. aureus* (MRSA) in Taif, Saudi Arabia, and assess the relationship between the phenotypic antimicrobial sensitivity patterns and the genes responsible for resistance. In addition, we examined the antimicrobial efficiency and application of silver nanoparticles (AgNPs) against MRSA isolates. Seventy-two nasal swabs were taken from patients; MRSA was cultivated on Mannitol Salt Agar supplemented with methicillin, and 16S rRNA sequencing was conducted in addition to morphological and biochemical identification. Specific resistance genes such as *ermAC*, *aacA-aphD*, *tetKM*, *vatABC* and *mecA* were PCR-amplified and resistance plasmids were also investigated. The MRSA incidence was ~49 % among the 72 *S. aureus* isolates and all MRSA strains were resistant to oxacillin, penicillin, and cefoxitin. However, vancomycin, linezolid, teicoplanin, mupirocin, and rifampicin were effective against 100% of MRSA strains. About 61% of MRSA strains exhibited multidrug resistance and were resistant to 3-12 antimicrobial medications (MDR). Methicillin resistance gene *mecA* was presented in all MDR-MRSA strains. Most MDR-MRSA contained a plasmid of > 10 kb. To overcome bacterial resistance, AgNPs were applied and displayed high antimicrobial activity and synergistic effect with penicillin. Our findings may help establish programs to control bacterial spread in communities as AgNPs appeared to exert a synergistic effect with penicillin to control bacterial resistance.

## Introduction

*Staphylococcus aureus* causes diseases ranging from fairly minor skin and soft-tissue infections to dangerous sepsis [1, 2]. All penicillins are ineffective against MRSA, including cephalosporins, carbapenems, semi-synthetic penicillinase-resistant congeners, and penems. Once methicillin was discovered in 1960, resistant strains were named after the showing of clinical isolates in England by 1967. MRSA was described in several countries primarily in the early 1980s. MDR-MRSA has been reported in numerous countries and is presently endemic in many hospitals worldwide, and commonly in developing countries such as Brazil [3].

Because of the increased mortality related to MRSA infections, the emergence of strains resistant to methicillin and other antimicrobials has become a major concern, particularly in the hospital context [2]. Between 1999 and 2002, increases in methicillin resistance were found in *S. aureus* isolates in European nations, namely Belgium, Germany, Ireland, the Netherlands, and the United Kingdom. In Northern, Western, and Southern Europe, MRSA incidence ranged from 1% to 40%, respectively [4].

The need to halt the spread of MRSA and decrease the incidence of MRSA infections in hospital settings now appears critical, as the multidrug-resistant organism’s prevalence persists. According to a recent study, the average MRSA transporter ratio among healthcare workers is 4.6%, with 5.1% having typical MRSA infections. MRSA levels must also be controlled by healthcare staff [2].

Investigation is a major and widely acknowledged tool for healthcare institutions to monitor the occurrence of illness due to multidrug-resistant bacteria and, if needed, to strengthen infection control operations [5, 6]. Furthermore, investigation of MRSA is a means of classifying occupied or contaminated patients for whom definite governor procedures may be employed. The employment of a plan of active investigation principles alongside interaction protections is recommended by several nations as an approach to stopping the nosocomial diffusion of MRSA [7].

MRSA is the mostly widely spread, multidrug-resistant pathogen triggering nosocomial infections in Europe. Assessments show that there are around 170,000 MRSA infections in European healthcare organizations every year, resulting in more than 5,000 mortalities, more than 1 million additional inpatient days, and extra charges of about €380 million [8]. Nevertheless, for a number of years, numerous countries have realized achievements in the anticipation and control of healthcare-associated MRSA (HA-MRSA) infections. New MRSA pools in hospitals are recognized in addition to community-associated MRSA (CA-MRSA) infections among the general population [9].

Data concerning the prevalence and mechanisms of some antimicrobial resistance have not been reported previously in the MRSA isolates from Taif, Saudi Arabia. Additionally, the multi-site action of nanosilver particles makes them a strong competitor for overcoming microbial resistance [10]. They are the most often investigated nanoparticles in nanobiotechnology due to their distinct chemical, physical, and biological features. The synergistic effect of antibiotic combined with AgNPs, especially penicillin, exerts significant antibacterial activity against pathogenic strains [11].

As a result, this work was conducted to examine the incidence of MRSA in the Taif area. Our study also assesses the relationship between phenotypic antimicrobial susceptibility patterns and resistance genes. Moreover, we sought to examine the incidence of streptogramin, aminoglycoside macrolide, tetracycline and lincosamide resistance genes amongst MRSA strains. Genes included in the current study were: *mecA*, *aacA-aphD*, *tetK*, *tetM*, *erm*(A), *erm*(C), *vat*(A), *vat*(B), and *vat*(C). Lastly, we investigated the antimicrobial activity and synergistic effect of AgNPs with penicillin, as well as their application to overcome bacterial infections.

## Materials and Methods

### Collection of Samples

In Taif Province, 72 nasal samples were collected from patients. Samples were sent directly to the laboratory and processed immediately or refrigerated at 4°C. AgNPs were those as obtained in our previous study [12].

### Isolation and Characterization of MRSA

The collected samples were cultured on Mannitol Salt Agar (MSA) by streaking. The cultures were incubated at 37°C for 24-48 h. The yellow *Staphylococcus* colonies exhibiting mannitol fermentation were chosen and subsequently characterized. Suspect colonies were kept at -70°C in 40% glycerol. MRSA was cultivated on MSA supplemented with methicillin (5 mg/l) to separate it from methicillin-sensitive *Staphylococcus aureus* (MSSA). MRSA was isolated from clinical specimens using MSA and methicillin [13]. After an incubation time of 24 h at 37°C, the morphological and biochemical properties of the bacterial strains were determined. Bergey's Manual of Systematic Bacteriology was used to describe the strains [14].

### Antimicrobial Susceptibility Assay

Kirby Bauer Test (disc diffusion assay) was employed [15]. Mueller Hinton agar (MHA) was plated with a suspension of *S. aureus* containing 1.5 × 108 cfu/ml. Following that, 6 mm discs with varied doses (2-30 μg) of antibiotics (OXOID, UK) were put onto the MHA surface and incubated for 24 h in a 37°C incubator. An inhibition zone represented susceptibility, and no zone indicated total bacterial resistance. For MIC detection, the antimicrobial agent (e.g., 1, 2, 4, 8, 16, 32 and 320 μg/ml) in a liquid growth medium was dispensed in a 96-well, micro-titer plate. The dilution was adjusted to a standardized microbial suspension at 0.5 on the McFarland scale, and placed in each well. After thorough mixing, the microtitration plate was incubated at 37°C for 24 h, and finally, the MICs were detected. To establish whether the isolate was resistant, intermediate, or susceptible, the MICs were linked to known MIC values of CLSI-defined breakpoints [15, 16]. The following antimicrobial agents were used: clindamycin (DA), erythromycin (E), gentamicin (GM), fosfomycin (FOS), penicillin (P), oxacillin (OX), cefoxitin (FOX), tobramycin (TOB), levofloxacin (LVX), moxifloxacin (MXF), mupirocin (MUP), linezolid (LNZ), teicoplanin (TEC), vancomycin (VA), tetracycline (TE), tigecycline (TGC), nitrofurantoin (NIT), fusidic acid (FA), rifampicin (RA), and sulfamethoxazole/trimethoprim (SXT).

## Molecular Characterization of MRSA

### Isolation of DNA

A Genomic DNA Purification Kit (Thermo Scientific, GeneJET, USA) was used with some modifications. Electrophoresis on 0.7% agarose gel with ethidium bromide was used to evaluate DNA samples. As a size standard, a molecular weight marker and a 1Kb DNA Ladder RTU (Ready-to-Use, GeneDireX, USA) were utilized.

### Detection of Resistance Genes by PCR

PCR was used to assess the presence of genes resistant to methicillin (*mecA*), aminoglycosides (*aacA-aphD*), tetracycline (*tetKM*), streptogramin A (*vatABC*), and macrolide-lincosamide-streptogramin B (*ermAC*). Also, 16S rDNA and *S. aureus*-specific sequences were determined as amplification control. Macrogen provided the primer set utilized for each gene mentioned in Table 1. Promega's GoTaq Green Master Mix Kit (USA) was utilized. PCR mixes were achieved in a total volume of 25 μl, including 12.5 μl Master Mix, 2 μl forward primer (10 pM), 2 μl reverse primer (10 pM), DNA template 2 μl, and 6.5 μl water.

The following amplification processes were performed using a DNA thermocycler (Labnet International, model: Multigene Opti Max): Three min at 95°C, 35 cycles each consisting of 30 s at 94°C, 30 s at 55°C, and 30 s at 72°C, followed by a ﬁnal extension step of 4 min at 72°C. Ampliﬁed samples were analyzed in 1% agarose gel and stained by ethidium bromide. A molecular weight marker and 100 bp DNA Ladder RTU (Ready-to-Use, GeneDireX), were used as a size standard.

### Isolation of Plasmid DNA

A GeneJET Plasmid Miniprep Set (Thermo Scientific, USA) was used with some modifications. Electrophoresis in 0.7% agarose gel was used to identify plasmid preparations, which were stained with ethidium bromide. As a size standard, a molecular weight marker and a 1Kb DNA Ladder RTU (Ready-to-Use, GeneDireX) were utilized.

### 16S rRNA Gene Analysis

One microliter of template DNA was added in 20 μl of PCR reaction solution. The next two primers were used: 27F-AGAGTTTGATCMTGGCTCAG and 1492R-TACGGY TACCTTGTTACGACTT. A total of 35 amplification cycles were completed at 94°C (45 s), 55°C (60 s), and 72°C (60 s). The length of the amplified DNA fragments was 1,400 bp. PCR products were electrophoresized in agarose gel (1%) and stained with ethidium bromide. As a size standard, a molecular weight marker and a 1Kb DNA Ladder RTU (Ready-to-Use, GeneDireX) were utilized. The two primers 518F-CCAGCAGCCGCGGTAATACG and 800R-TACCAGGGTATCTAATCC were used for 16S rRNA sequencing. An automated DNA sequencing machine (Applied BioSystems, USA) was employed to identify sequencing products. To prepare a phylogenetic tree, selected sequences of different microorganisms with the highest similarity to the 16S rRNA sequences of bacterial isolates were retrieved from the gene bank and aligned using CLUSTAL W (1.81) Multiple Sequence Alignment.

### Determination of MIC and MBC of Silver Nanoparticles

A 96-well microtitration plate was employed for testing, and 200 μl of AgNPs (30 μg/ml) or AgNPs with penicillin (10 μg/ml; 1:1) were piped into the six wells (except the first and last) in column 1 (far left side of the plate). The wells in each row were then filled with 100 μl of broth medium, which was adequate for bacterial growth. After that, 100 μl of tested solution was collected from column 1 and serially diluted till column 10. With the exception of column 10, which served as a control, the tested bacteria were then injected into each well containing their respective medium. The 96-well plate was then incubated for 24 h at 37°C. The MBC of AgNPs is defined as the lowest concentration of an antimicrobial agent killing the majority (99.99%) of bacterial inoculums. If the antimicrobial agent were to be withdrawn, the bacteria would likely start to grow again because the MIC of nanosilver relates to its inhibitory activity.

### Antimicrobial Activity of AgNPs Using Well Agar Diffusion Method

Using the well agar diffusion technique, the AgNPs were displayed for antibacterial efficiency against strains of tested bacteria [17]. The multidrug-resistant microorganisms were first cultured in nutrient broth at 37°C. The overnight developed cultures were then sub-cultured for 2 h in nutrient broth medium until they reached 0.01 OD. Following that, 100 μl of each culture was equally placed onto nutrient agar plates. The penicillin, AgNPs, and AgNPs with penicillin were put into the agar wells. The plates were incubated for 24 h at 37°C. The bacterial clearance diameter was applied to compute the inhibition zone.

## Results 

### Isolation and Characterization of *S. aureus*

Nasal swab samples were collected from 72 individuals. By direct plating on Mannitol Salt Agar with methicillin, 34 (47%) of the 72 *S. aureus* isolates tested positive for MRSA. The MRSA isolates were designated as NA05, NA07, NA09, NA12, NA14, NA24, NA30, NA31, NA36, NA38, NA40, NA44, NA45, NA48, NA67, NA73, NA76, NA77, NA78, NA80, NA81, NA82, NA83, NA85, NA92, NA93, NA94, NA97, NA101, NA102, NA107, NA109, NA110 and NA111. All isolates were identified as *S. aureus* both morphologically and biochemically (Table 2). *S. aureus* colonies on blood agar are white or golden. Catalase, an enzyme that transforms hydrogen peroxide (H_2_O_2_) to water and oxygen, is produced by all the strains. Staphylococci, enterococci, and streptococci may all be distinguished with the catalase test. Among other things, the anaerobic fermentation of mannitol and trehalose by the majority of these strains can be utilized to pinpoint *S. aureus*. Plasma clotting may also be triggered by *S. aureus*.

### Antimicrobial Susceptibility Test

This test was performed on 72 isolates of *S. aureus* obtained from nasal swabs. The MICs of these bacterial isolates were evaluated against various drugs (Table 3). The acquired data were revealed using CLSI 2015 rules. Tables 4 demonstrate that 47% of the isolates were MRSA isolates. Cefoxitin, oxacillin, and penicillin were all resistant to all MRSA strains. However, all MRSA isolates tested positive for teicoplanin, linezolid, vancomycin, mupirocin, and rifampicin. In this work, the MRSA prevalence was found to be 47%. MRSA antibacterial resistance pattern is shown in Table 4. MRSA was completely resistant to 3-12 antibacterial drugs. Sixty-one percent of MRSA strains from nasal swabs were MDR. NA05, NA24, NA31, NA40, NA45, NA67, NA73, and NA93 MRSA strains were resistant to 11, 12, 11, 12, 7, 6, 12, and 10 antimicrobial agents, respectively. As a result, most of the MDR-MRSA isolates were chosen for future research. The MDR strain incidence among MRSA was revealed to be relatively high (> 90%) in the current investigation.

### Antimicrobial Resistance Genes

Antimicrobial resistance genes were examined in MDR-MRSA isolates NA05, NA24, NA31, NA40, NA45, NA67, NA73, and NA93 (resistant to 5 or more antimicrobial agents). First, the chromosomal DNA from these samples was extracted. The occurrence of sufficient DNA for the PCR reaction was confirmed by 0.7% agarose gel electrophoresis in a volume of 5 μl of the preparation (data not shown). The PCR was applied to look for genes related to methicillin resistance (*mecA*), aminoglycosides (*aacA-aphD*), tetracycline (*tetKM*), streptogramin A (*vatABC*), and macrolide-lincosamide-streptogramin B (*ermAC*) (Table 5). As an amplification control, the 16S rDNA and *S. aureus*-specific sequences were identified. These genes were amplified using PCR. A volume of 5 μl of each PCR reaction was examined using 0.7% agarose gel electrophoresis, which verified that the PCR products were of the predicted sizes. The 16S rDNA and *S. aureus*-specific sequences were found in all isolates, according to these findings. Methicillin resistance genes (*mecA*) were found in isolates NA05, NA24, NA31, NA40, NA45, NA67, NA73, and NA93. Methicillin (*mecA*), aminoglycoside (*aacA-aphD*), tetracycline (*tetM*), and macrolide-lincosamide-streptogramin B (*ermA*) resistance genes were found in isolates NA05, NA24, NA31, NA40, NA73, and NA93. NA45 included resistance genes for methicillin (*mecA*), aminoglycosides (*aacA-aphD*), tetracycline (*tetK*), and macrolide-lincosamide-streptogramin B (*ermA*). The strain NA67, on the other hand, only possessed the methicillin resistance gene (*mecA*). The *mecA* gene was detected in all MRSA isolates in this study, demonstrating that all of the isolates were MRSA.

### Antimicrobial Resistance Plasmids

Plasmids were identified from the majority of MDR-MRSA isolates recovered from the nose, including NA05, NA24, NA31, NA40, NA45, NA67, NA73, and NA93. Moreover, 0.7% agarose gel electrophoresis was performed on a volume of 15 μl of each plasmid preparation. Table 6 shows that all isolates contained 1-3 plasmids. Most isolates had plasmids larger than 10 kb. One plasmid larger than 10 kb was found in isolates NA05, NA40, NA45, and NA93.

### 16S rRNA Analysis and Phylogenetic Tree

**Fig. 1:**
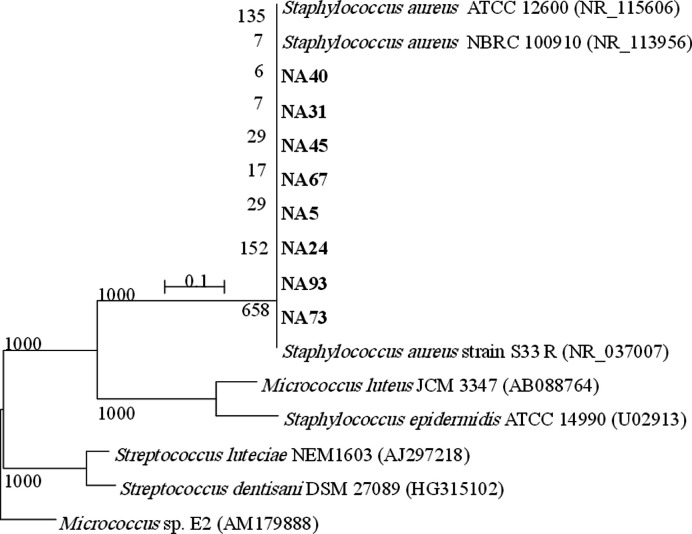
A phylogenetic tree of MRSA isolates from nasal swabs based on the nucleotide sequences of 16S rRNA genes was constructed by neighbor-joining method. The scale bar shows the genetic distance. The number presented next to each node shows the percentage bootstra*p* value of 1,000 replicates. The GenBank accession numbers of the bacteria are presented in parentheses.

MRSA isolates NA05, NA24, NA31, NA40, NA45, NA67, NA73, and NA93 were PCR-amplified (1,500 bp) and sequenced for further characterization. The 16S rRNA gene sequences of the MDR-MRSA isolates from nose were deposited in the DDBJ/EMBL/GenBank nucleotide sequence databases, with the following accession numbers: LC107786 (*S. aureus* NA5), LC107787 (*S. aureus* NA24), LC107788 (*S. aureus* NA31), LC107789 (*S. aureus* NA40), *S. aureus* NA45), LC107791 (*S. aureus* NA73), LC107792 (*S. aureus* NA67), and LC107793 (*S. aureus* NA93). The 16S rRNA sequences of MDR isolates were compared to sequences already present in the gene bank. Fig. 1 displays a dendrogram with the findings of the 16S rRNA investigation. The isolates NA05, NA24, NA31, NA40, NA45, NA67, NA73, and NA93 best matched the *Staphylococcus* group. According to their 16S rRNA sequences, the *S. aureus* isolates are most closely related to *S. aureus*. These conclusions are supported by the results of the morphological and biochemical characterizations. The 16S rRNA gene of the *S. aureus* isolates NA05, NA24, NA31, NA40, NA45, NA67, NA73, and NA93 is 99 percent similar. According to these findings, the isolates (NA05, NA24, NA31, NA40, NA45, NA67, NA73 and NA93) are new *S. aureus* isolates.

### Antimicrobial Activity and MIC of AgNPs and Their Combination

**Fig. 2:**
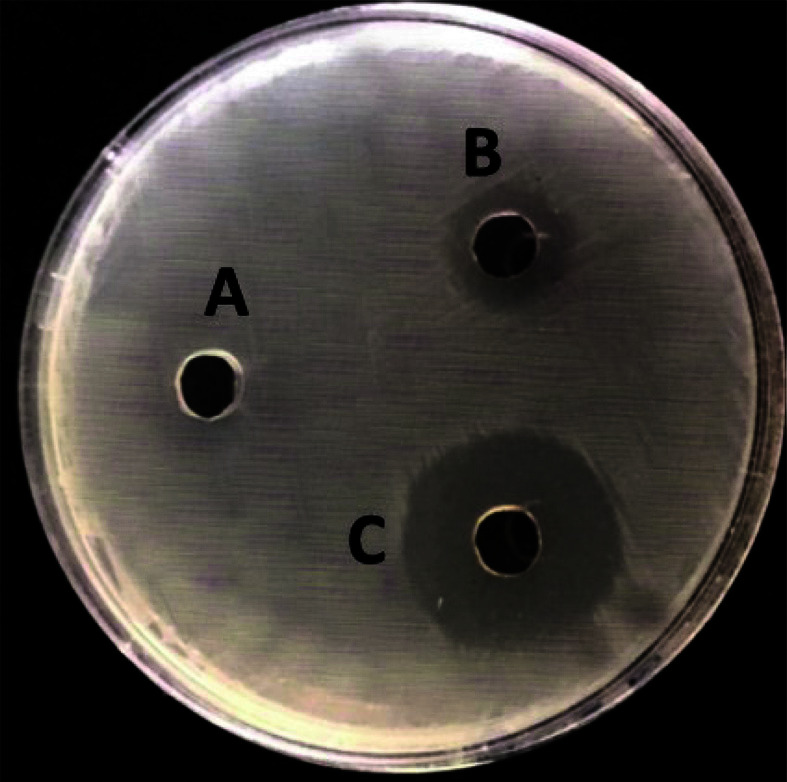
Antibacterial activity of penicillin (A), AgNPs (B), AgNPs/penicillin (C) against *S. aureus* (MRSA) using the agar diffusion method.

The antimicrobial properties of synthetic AgNPs against tested strains were investigated. According to Table 6, AgNPs were successful at preventing the growth of the MDR bacteria that were tested. The MIC range of the AgNPs was 9-12 μg/ml, whereas the MIC range of AgNPs with penicillin was 4-6 μg/ml. Therefore, AgNPs and penicillin together increased antibacterial efficiency by almost 2-3 times. Significant antibacterial effectiveness against the studied microorganisms was increased by combining AgNPs with penicillin (Table 7, Fig. 2).

## Discussion

Because it may clot extracellular plasma, *S. aureus* can be differentiated from the bulk of other staphylococci [18, 19]. The 72 *S. aureus* isolates that we analyzed in the current study had nasal carriage of MSSA and MRSA in 53% and 47% of individuals, respectively. The proportions of *S. aureus* nasal carriage agree with other studies from China (23.1%) and Taiwan (19.3%) [20, 21]. Although the MRSA frequency in the Australian research was higher than that in comparable studies in Taiwan (2.2%) and the US (2.0%), the study revealed no indication of MRSA carriage [21, 22]. Some articles claim that the variation might be explained by the differences in environmental conditions, the intensity of adverse environmental interactions, and the regulations in place in different countries for the prevention and control of infection. However, in contrast to the general population, the rates among health care employees were as high as 6.2-9.7% [23]. Seventy-two *S. aureus* isolates that were obtained from nasal swabs were evaluated for antibiotic susceptibility. All MRSA strains tested positive for sensitivity to linezolid, teicoplanin, vancomycin, mupirocin, and rifampicin and 100% resistance to oxacillin, penicillin, and cefoxitin. In this investigation, the MRSA incidence was found to be 47%, which is lower than the rates of 63.20, 75, and 90%[24-26] reported in other studies. Nevertheless, according to various other studies, the MRSA prevalence is modest and varies widely across different nations [27-29], from 5.3% to 38.1%. The differences between hospitals may be due to different identification strategies, effective infection control methods, healthcare services, and antibiotic usage.

With the exception of linezolid, teicoplanin, vancomycin, mupirocin, and rifampicin, MRSA isolates were found to be more resistant to all antimicrobials than MSSA strains. Despite being the most effective antibacterial against MRSA isolates, linezolid's high cost restricts its use in therapy. Resistance to 3-11 antimicrobials was recorded in the case of all MRSA strains. Resistance to one antimicrobial as a minimum in three or more classes was characterized as multidrug-resistant (MDR) [30]. Sixty-one percent of MRSA isolates from nasal swabs were MDR. MRSA isolates NA05, NA24, NA31, NA40, NA45, NA67, NA73 and NA93 were resistant to 10, 11, 10, 11, 6, 5, 11 and 9 antimicrobial agents, respectively. As a result, the majority of MDR-MRSA isolates were chosen for additional research. The MRSA strain incidence was found in our investigation to be relatively high (> 90%).

The MRSA strain incidence has varied from 80% to 100% in several earlier reports from other nations [31, 32]. This high incidence might be due to numerous reasons like elongated hospitalization, nasal bearing of MRSA, abuse of multiple antibiotics, unsuccessful control procedures and deficiency of consciousness amongst hospital workers. In Doon Valley, Uttrakhand, the MRSA prevalence among patients was assessed using a survey [33]. Following established methodology, a bacteriological analysis of 300 nose swabs was displayed. Isolates were verified by using the DNAse test, Gram staining, coagulase positivity, and mannitol fermentation. Thirty-eight (38%) of the 111 persons with *S. aureus* strains were methicillin resistant [33].

Patients in the dialysis ward (55.5%) had the highest MRSA colonization rate, followed by those in the burn unit (32.5%) and general medical ward (22.7%). The analysis also revealed that the use of new antibiotics was the primary contributing reason to the MRSA development. This study's high MRSA carriage rate suggests the need for widespread infection management to stop the disease spread [33]. There have been several proposals for MRSA detection in clinical specimens [34]. These may be attributed to the increase in *S. aureus* contact and subsequent acquisition risk in the hospital setting. According to a recent study, MRSA that was isolated from nasal swabs and tested for antibiotic sensitivity showed that multidrug resistance was common [35]. All isolates included the *mecA* gene, which confers methicillin resistance. Tetracycline (*tetM*), methicillin (*mecA*), aminoglycosides (*aacA-aphD*), and macrolide-lincosamide-streptogramin B (*ermA*) resistance genes were often found in isolates NA05, NA24, NA31, NA40, NA73, and NA93. Methicillin (*mecA*), aminoglycoside (*aacA-aphD*), tetracycline (*tetK*), and macrolide-lincosamide-streptogramin B resistance genes occurred in strain NA45 (*ermA*). The isolate NA67, however, was limited to having the methicillin resistance gene (*mecA*). All of the MRSA strains in our investigation carried the *mecA* gene, proving that each isolate belonged to the genus. Previous studies discovered that 62 (92.53%) of 67 MRSA isolates tested positive for *mecA*, while 7.46% of the remaining oxacillin-resistant isolates (which tested negative for *mecA*) must be MRSA due to some other reasons [36].

Each isolate had one to three plasmids. In the majority of isolates, a plasmid larger than 10 kb was found. Each of the isolates NA05, NA40, NA45, and NA93 had one plasmid with a size more than 10 kb. Plasmid profiles have been mentioned as one technique for categorizing MRSA and MSSA [37]. The MRSA strains that were employed in this study were all plasmid-carrying. Plasmids were found in every isolate, indicating that plasmid profile analysis has a very high level of justifiable competence when looking at the epidemiology of MRSA. Plasmid characterization of *S. aureus* strains reveals a wide variety of plasmid sizes and a reliable correlation between plasmid profiles and resistance traits. The bacteria also showed resistance to at least six different kinds of antibiotics or more as a result of harboring different plasmids. Nineteen non-multidrug-resistant (NMR) isolates were only found to be resistant to methicillin in a previous investigation, but all NMR strains harboring at least one or more plasmid were also shown to be resistant to one other antibiotic. The MDR phenotype from HA-MRSA is not associated with enhancing plasmid carriage and is characterized by a lack of plasmid DNA, despite the fact that plasmid carriage exhibits considerable responsibility in antimicrobial resistance, particularly in NMR-HA-MRSA and CA-MRSA [38].

AgNPs' antimicrobial activity has been reported and explained to have a synergistic effect with penicillin, boosting antibacterial efficiency by roughly 2-3 times, which is comparable with the findings of Gad El-Rab *et al*.[39]. Furthermore, AgNPs show antibacterial effectiveness against multidrug-resistant infections, have simultaneous modes of action, and have a synergistic effect against pathogens such as *S. aureus* when paired with antibiotics [40]. Significant antibacterial effectiveness against tested bacteria was increased by increasing the concentration of AgNPs alone or in combination with penicillin. These data are consistent with the results of Gad El-Rab *et al*. [39], who discovered that adding AgNPs to antibiotics boosted and regenerated antibiotic activity. Furthermore, the strong antibacterial efficiency is linked to tiny nanoparticle penetration and cell membrane disruption, which leads to microbial cell death [41]. Nanosilver's mechanism of action on bacteria has been demonstrated to be largely due to membrane permeability loss, which leads to leakage of cellular components, followed by penetration into the inner membrane and eventually disrupting respiratory chain dehydrogenases, preventing bacterial growth.

Among patients, MRSA (47%) was more common than MSSA (53%). MRSA showed no resistance to cefoxitin, oxacillin, or penicillin. However, it was discovered that the usage of linezolid, teicoplanin, vancomycin, mupirocin, and rifampicin was 100 percent effective against all MRSA strains. All MRSA had antimicrobial drug resistance to 3-11 drugs, while 61% of MRSA cases involved MDR. The majority of MDR-MRSA isolates had drug resistance to 5-11 different antibacterials. Every single isolate of MDR-MRSA included methicillin resistance genes (*mecA*). Methicillin (*mecA*), aminoglycosides (*aacA-aphD*), macrolide-lincosamide-streptogramin B (*ermA*), tetracycline (*tetM*), macrolide-lincosamide-streptogramin B (*ermA*), and other antibiotics were all to some extent resistant in isolates NA05, NA24, NA31, NA40, NA73, and NA93. The isolate NA45 contained the genes associated with methicillin resistance (*mecA*), aminoglycoside (*aacA-aphD*), tetracycline (*tetK*), and macrolide-lincosamide-streptogramin B (*ermA*). However, the isolate NA67 contained only the gene associated with methicillin resistance (*mecA*). Almost all MDR-MRSA isolates from nasal swabs contained 1-3 plasmids. A plasmid of >10 kb occurred in most isolates. Also, AgNPs exhibited high antimicrobial activity and synergistic effect with penicillin against MRSA strains and can therefore be used as a nano-drug for overcoming bacterial infections.

## Figures and Tables

**Table 1:** The primer sequences and predicted sizes used in the PCR.

Target gene	Oligonucleotide sequence (5'-3')	Amplicon size (bp)
16S rDNA	16Sf: CAG CTC GTG TCG TGA GAT GT	420
	16Sr: AAT CAT TTG TCC CAC CTT CG	
*S. aureus*-specific sequence	sauf: AATCTTTGTCGGTACACGATATTCTTCACG	107
	saur: CGTAATGAGATTTCAGTAGATAATACAACA	
*mecA*	mecAf: AAA ATC GAT GGT AAA GGT TGG C	532
	mecAr : AGT TCT GCA GTA CCG GAT TTG C	
*aacA-aphD*	aacA-aphDf: TAA TCC AAG AGC AAT AAG GGC	227
	aacA-aphDfr: GCC ACA CTA TCA TAA CCA CTA	
*tetK*	tetKf: GTA GCG ACA ATA GGT AAT AGT	360
	tetKr: GTA GTG ACA ATA AAC CTC CTA	
*tetM*	tetMf: AGT GGA GCG ATT ACA GAA	158
	tetMr: CAT ATG TCC TGG CGT GTC TA	
*vatA*	vatAf: TGG TCC CGG AAC AAC ATT TAT	268
	vatAr: TCC ACC GAC AAT AGA ATA GGG	
*vatB*	vatBf: GCT GCG AAT TCA GTT GTT ACA	136
	vatBr: CTG ACC AAT CCC ACC ATT TTA	
*vatC*	vatCf: AAG GCC CCA ATC CAG AAG AA	467
	vatCr: TCA ACG TTC TTT GTC ACA ACC	
*ermA*	ermAf: AAG CGG TAA ACC CCT CTG A	190
	ermAr: TTC GCA AAT CCC TTC TCA AC	
*ermC*	ermCf: AAT CGT CAA TTC CTG CAT GT	299
	ermCr: TAA TCG TGG AAT ACG GGT TTG	

**Table 2:** Morphological and biochemical characteristics of MRSA isolates from nasal swabs.

Characteristics	MRSA isolates
Gram's stain	+
Cocci	+
Urease	+
Nitrate Reduction	+
DNase Production	+
Urease	+
Motility	-
Catalase test	+
Coagulase test	+
Oxidase test	-
Indole test	-
Methyl red	+
Voges-proskaure	+
Citrate	+
H_2_S Production	-
Fermentation Glucose	+
Trehalose	+
Lactose	+
Mannitol	+
Sucrose	+
Maltose	+
Probable bacteria	*S. aureus*

+, positive; -, negative

**Table 3:** The MIC values of MRSA isolates from nasal swabs.

Isolates	MIC values (μg/ml)																			

	P	OX	FOX	GM	TOB	LVX	MXF	E	DA	LNZ	TEC	VA	TE	TGC	FOS	NIT	FA	MUP	RA	SXT
NA05	≤0.5	≤4	≤8	16	16	4	1	8	8	1	0.5	2	16	0.12	8	16	32	8	0.5	320
NA07	≤0.5	≤4	≤8	0.5	1	0.25	0.25	0.25	0.25	2	0.5	1	1	0.12	8	16	16	2	0.5	10
NA09	≤0.5	≤4	≤8	0.5	1	0.25	0.25	0.25	0.25	2	0.5	0.5	16	0.25	8	16	8	2	0.5	10
NA12	≤0.5	≤4	≤8	4	2	4	1	0.25	0.25	2	0.5	0.5	1	0.12	8	16	0.5	2	0.5	10
NA14	≤0.5	≤4	≤8	0.5	1	0.25	0.25	8	0.25	2	0.5	0.5	1	0.12	8	32	1	2	0.5	160
NA24	≤0.5	≤4	≤8	16	16	8	2	8	8	2	0.5	1	16	0.25	8	16	32	8	0.5	320
NA30	≤0.5	1	≤8	0.5	1	0.12	0.25	0.25	0.25	2	0.5	0.5	16	0.12	8	16	8	2	0.5	10
NA31	≤0.5	≤4	≤8	16	16	4	1	8	8	1	0.5	1	16	0.12	8	16	32	8	0.5	320
NA36	≤0.5	≤4	≤8	0.5	1	0.25	0.25	0.25	0.25	2	0.5	0.5	1	0.12	8	32	8	2	0.5	10
NA38	≤0.5	≤4	≤8	0.5	1	0.25	0.25	0.25	0.25	2	0.5	0.5	1	0.12	8	32	0.5	2	0.5	10
NA40	≤0.5	≤4	≤8	16	16	8	4	8	8	2	0.5	0.5	16	0.12	64	32	32	2	32	10
NA44	≤0.5	≤4	≤8	0.5	1	0.12	0.25	0.25	0.25	2	0.5	2	1	0.12	8	32	0.5	2	0.5	10
NA45	≤0.5	≤4	≤8	0.5	1	0.12	0.25	8	0.25	2	4	0.5	16	0.25	64	16	32	2	0.5	10
NA48	≤0.5	≤4	≤8	0.5	1	0.12	0.25	0.25	0.25	2	0.5	0.5	1	0.12	8	16	0.5	2	0.5	10
NA67	≤0.5	≤4	≤8	0.5	1	8	2	0.25	0.25	2	0.5	0.5	1	0.12	8	32	0.5	2	0.5	320
NA73	≤0.5	≤4	≤8	16	16	8	2	8	8	1	0.5	0.5	16	0.12	8	16	32	8	0.5	320
NA76	≤0.5	≤4	≤8	0.5	1	0.25	0.25	8	0.25	2	0.5	2	1	0.12	8	32	0.5	2	0.5	80
NA77	≤0.5	≤4	≤8	0.5	1	0.12	0.25	0.25	0.25	2	0.5	2	1	0.12	8	16	8	2	0.5	10
NA78	≤0.5	≤4	≤8	0.5	1	0.12	0.25	8	0.25	1	0.5	1	1	0.12	8	16	1	2	0.5	20
NA80	≤0.5	≤4	≤8	0.5	1	0.25	0.25	0.25	0.25	2	0.5	1	1	0.12	8	16	8	2	0.5	10
NA81	≤0.5	≤4	≤8	0.5	1	0.12	0.25	0.25	0.25	2	0.5	0.5	1	0.12	8	32	0.5	2	0.5	10
NA82	≤0.5	≤4	≤8	0.5	1	0.12	0.25	8	0.25	2	0.5	0.5	1	0.12	8	32	0.5	2	0.5	10
NA83	≤0.5	2	≤8	0.5	1	0.12	0.25	0.25	0.25	2	0.5	0.5	16	0.12	8	16	0.5	2	0.5	40
NA85	≤0.5	≤4	≤8	0.5	1	0.25	0.25	8	0.25	2	0.5	1	1	0.12	8	16	0.5	2	0.5	320
NA92	≤0.5	≤4	≤8	0.5	1	0.25	0.25	0.25	0.25	2	0.5	0.5	1	0.12	8	16	0.5	2	0.5	10
NA93	≤0.5	≤4	≤8	16	16	0.12	0.25	8	8	0.5	0.5	0.5	16	0.12	8	16	32	2	0.5	320
NA94	≤0.5	≤4	≤8	0.5	1	0.25	0.25	0.25	0.25	2	0.5	0.5	1	0.12	8	32	0.5	2	0.5	10
NA97	≤0.5	≤4	≤8	0.5	1	0.12	0.25	0.25	0.25	2	0.5	0.5	1	0.12	8	16	8	2	0.5	10
NA101	≤0.5	≤4	≤8	0.5	1	0.25	0.25	0.25	0.25	2	0.5	0.5	1	0.12	8	32	8	2	0.5	10
NA107	≤0.5	≤4	≤8	0.5	1	0.25	0.25	0.25	0.25	2	0.5	0.5	1	0.12	8	32	8	2	0.5	10
NA109	≤0.5	≤4	≤8	0.5	1	0.25	0.25	8	0.25	2	0.5	0.5	1	0.12	8	32	0.5	2	0.5	10
NA110	≤0.5	≤4	≤8	0.5	1	0.25	0.25	8	0.25	2	0.5	0.5	1	0.12	8	32	0.5	2	0.5	10
NA111	≤0.5	≤4	≤8	0.5	1	0.12	0.25	0.25	0.25	2	0.5	2	1	0.12	8	16	0.5	2	0.5	10
NA102	≤0.5	1	≤8	0.5	1	0.25	0.25	8	0.25	2	0.5	0.5	1	0.12	8	32	8	2	0.5	10

*Penicillin P, oxacillin OX, cefoxitin FOX, gentamicin GM, tobramycin TOB, levofloxacin LVX, moxifloxacin MXF, erythromycin E, clindamycin DA, linezolid LNZ, teicoplanin TEC, vancomycin VA, tetracycline TE, tigecycline TGC, fosfomycin FOS, nitrofurantoin NIT, fusidic Acid FA, mupirocin MUP, rifampicin RA and sulfamethoxazole/trimethoprim SXT.

**Table 4:** Antimicrobial resistance patterns for MRSA isolates from swabs.

Isolates	Antimicrobial resistance patterns	Total number
NA24	P,OX,FOX,GM,TOB,LVX,MXF,E,DA,TE,FA,SXT	12
NA40	P,OX,FOX,GM,TOB,LVX,MXF,E,DA,TE,FOS,FA	12
NA73	P,OX,FOX,GM,TOB,LVX,MXF,E,DA,TE,FA,SXT	12
NA31	P,OX,FOX,GM,TOB,LVX,E,DA,TE,FA,SXT	11
NA05	P,OX,FOX,GM,TOB,LVX,E,DA,TE,FA,SXT	11
NA93	P,OX,FOX,GM,TOB,E,DA,TE,FA,SXT	10
NA45	P,OX,FOX,E,TE,FOS,FA	7
NA14	P,OX,FOX,E,DA,SXT	6
NA67	P,OX,FOX,LVX, MXF,SXT	6
NA76	P,OX,FOX,E,SXT	5
NA82	P,OX,FOX,E,DA	5
NA85	P,OX,FOX,E,SXT	5
NA77	P,OX,FOX,FOX	5
NA109	P,OX,FOX,E,DA	5
NA110	P,OX,FOX,E,DA	5
NA102	P,OX,FOX,E,DA	5
NA09	P,OX,FOX,TE	4
NA12	P,OX,FOX,LVX	4
NA30	P,OX,FOX,TE	4
NA78	P,OX,FOX,E	4
NA83	P,OX,FOX,TE	4
NA92	P,OX,FOX	3
NA07	P,OX,FOX	3
NA36	P,OX,FOX	3
NA38	P,OX,FOX	3
NA44	P,OX,FOX	3
NA48	P,OX,FOX	3
NA80	P,OX,FOX	3
NA81	P,OX,FOX	3
NA111	P,OX,FOX	3

*Penicillin P, oxacillin OX, cefoxitin FOX, gentamicin GM, tobramycin TOB, levofloxacin LVX, moxifloxacin MXF, erythromycin E, clindamycin DA, linezolid LNZ, teicoplanin TEC, vancomycin VA, tetracycline TE, tigecycline TGC, fosfomycin FOS, nitrofurantoin NIT, fusidic Acid FA, mupirocin MUP, rifampicin RA and sulfamethoxazole/trimethoprim SXT.

**Table 5:** The antibiotic resistance genes in MRSA isolates from nasal swabs.

Isolates	16S rRNA (420bp)	Sau (107bp)	Resistance genes								

			*mecA* (532bp)	*aaA-aphD* (227bp)	*tetK* (360bp)	*tetM* (158bp)	*VatA* (268bp)	*VatB* (136bp)	*VatC* (467bp)	*ermA* (190bp)	*ermC* (299bp)
NA05	+	+	+	+	-	+	-	-	-	+	-
NA24	+	+	+	+	-	+	-	-	-	+	-
NA31	+	+	+	+	-	+	-	-	-	+	-
NA40	+	+	+	+	-	+	-	-	-	+	-
NA45	+	+	+	+	+	-	-	-	-	+	-
NA67	+	+	+	-	-	-	-	-	-	+	-
NA73	+	+	+	+	-	+	-	-	-	+	-
NA93	+	+	+	+	-	+	-	-	-	+	-

+, positive; -, negative

**Table 6:** Plasmid profiles of MRSA isolates from nasal swabs.

Isolates	Plasmid patterns (kb)	Total number
NA05	>10kb	1
NA24	>1.5kb,4kb, >10kb	3
NA31	2kb,3kb,4kb	3
NA40	>10kb	1
NA45	>10kb	1
NA67	1.5kb,>10kb,>10kb	3
NA73	3kb,4kb, >10kb	3
NA93	>10kb	1

**Table 7:** Minimum inhibitory concentration (MIC) and maximum inhibitory concentration (MBC) of AgNPs against tested bacteria.

Isolates	AgNPs (μg/ml)			AgNPs/penicillin (μg/ml)		
	
	MIC	MBC	MBC/MIC	MIC	MBC	MBC/MIC
NA05	12	22	1.83	3	4	1.33
NA24	14	23	1.64	4	5	1.25
NA31	10	20	2	3	4.5	1.5
NA40	9	18	2	2	4	2
NA45	14	25	1.8	4	6	1.5
NA67	14	24	1.7	4	6	1.5
NA73	9	17	1.88	3	5	1.66
